# Supplementary material for: CSF circulation and dispersion yield rapid clearance from intracranial compartments
Source: Front Bioeng Biotechnol. 2022 Sep 12;10:932469. doi: 10.3389/fbioe.2022.932469 (PMC9510842; doi:10.3389/fbioe.2022.932469)
Supplement: Supplementary file 1 [file DataSheet1.ZIP › frontiers_SupplementaryMaterial.pdf]

# Supplementary Material

## 1 SUPPLEMENTARY DATA

### 1.1 Time resolution convergence

In Figure S1 we present the first 10 hours of simulation results from Model I using a time step of $\Delta t = 4000$, $\Delta t = 2000$ and $\Delta t = 1000$. The results shown in the Results section in our paper was obtained using $\Delta t = 4032$. The error always stayed below 3.7 % in the SAS and 6.5 % in the ISF relative to the peak concentration observed in model I.

### 1.2 Mesh resolution convergence

As mesh refinements of the entire geometry is too expensive, a mesh resolution study was performed on a partition of the mesh containing both CSF and brain tissue. The partition is a 30x40x30 mm cut of the front left of the brain (Supplementary Figure S2). The initial resolution of the partition was comparable to the resolution of the mesh used in the study simulations. The mesh was then refined to contain two and four times more cells compared to the initial resolution. First, the Stokes equation was solved as described in the Methods section, but except for a source $\nabla \cdot u = g$, we used a pressure boundary condition, $\mu \nabla u \cdot n - pn = p_0 n$ on $\partial\Omega_{\text{in}}$, on one of the CSF edges. $p_0 = 5 \cdot 10^{-7}$ Pa was adjusted to get a reasonable velocity of around 10 $\mu$m/s. We allowed free outflow, $\mu \nabla u \cdot n - pn = 0$ on $\partial\Omega_{\text{in}}$, on the opposite CSF edge. Calculating tracer transport was done in the same way as described in the Methods section, except for there being a constant concentration of $c = 0.1 \cdot$ mmol/L on $\partial\Omega_{\text{in}}$ and free flow $c = 0$on $\partial\Omega_{\text{out}}$. Tracer transport was simulated over a time period of 10 hours, and the maximal fluid velocity in the SAS and the maximal tracer concentration (after T = 10 hours) in the parenchyma were stored for comparison with finer meshes.

Table S1 shows how the maximum velocity and tracer concentration in the brain changes as mesh resolution is improved. The maximum velocity changes from 12.37 $\mu$m/s to 12.50 $\mu$m/s as the resolution increased fourfold. Similarly, the maximum tracer concentration in the ISF changes from $5.489$ to $5.335$

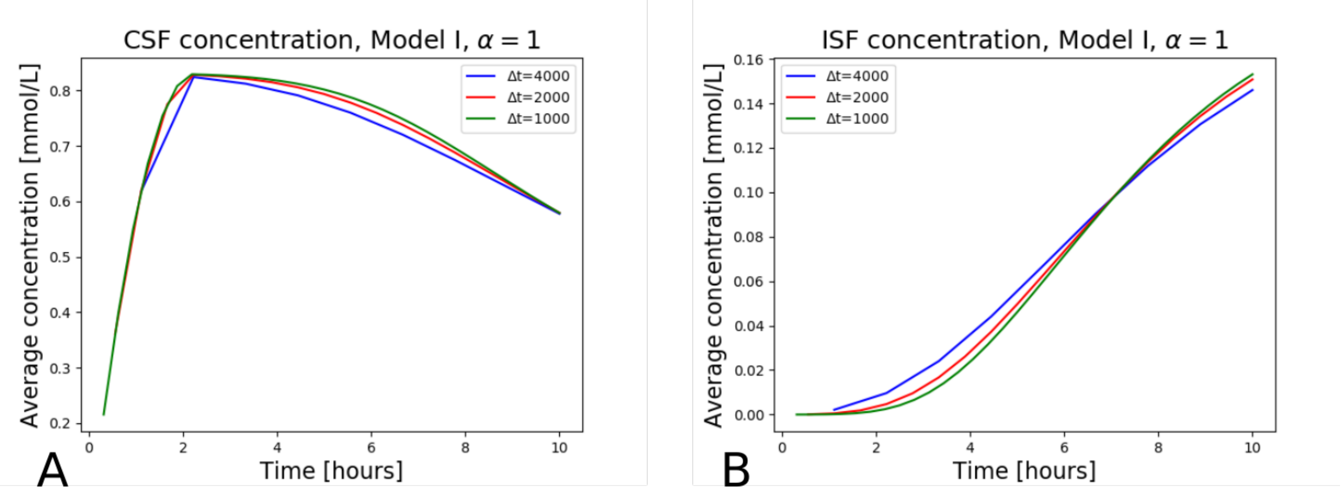


**Figure S1.** The figure shows the CSF and ISF average concentration in Model I with $\alpha = 1$ for the first 10 hours. Plotted are the results for three different time steps of increasing resolution.

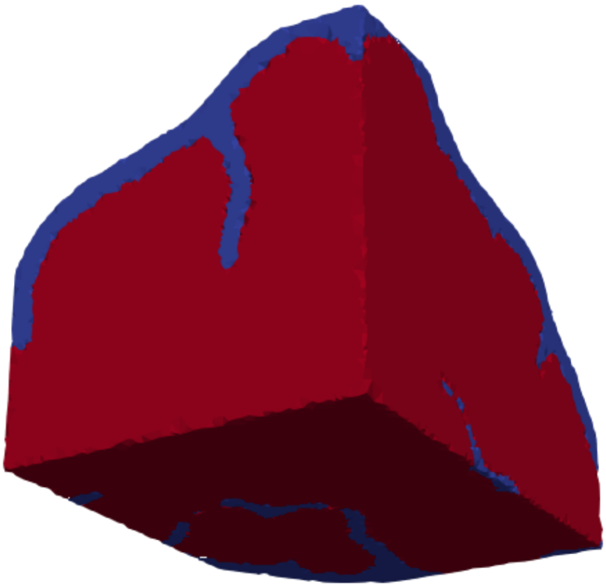

**Figure S2.** A 30x40x30 mm section of the brain mesh containing both the CSF and ISF space.

| Resolution | Maximum velocity [$\mu$m/s] | Maximum tracer concentration in the ISF [mmol/L] |
|---|---|---|
| Original | 12.37 | 5.489 |
| X2 | 12.48 | 5.421 |
| X3 | 12.50 | 5.335 |

**Table S1.** Convergence of maximum velocity and maximum tracer concentration in the ISF. Original, X2 and X4 denotes the original mesh resolution, 2 and 4 times the original mesh resolution respectively.

mmol/L. The relative error is thus, 1.04 % and 2.81 % between the coarsest and finest mesh for fluid velocity and tracer concentration respectively.
